# Rapid, effective and low-cost purification of dideoxy-sequencing reactions by home-made magnetic beads suspension and magnetic separator

**DOI:** 10.1371/journal.pone.0279432

**Published:** 2022-12-22

**Authors:** Hidenori Sassa, Kota Ikebe

**Affiliations:** Graduate School of Horticulture, Chiba University, Chiba, Japan; University of Pécs: Pecsi Tudomanyegyetem, HUNGARY

## Abstract

Removal of excess dideoxy terminators from the sequencing mix after the enzymatic reaction is a key process affecting the dideoxy/Sanger sequencing quality. Ethanol precipitation may be the most popular clean-up method because of its low cost; however, it takes a long centrifugation time and frequently results in low quality sequence data. Commercially available clean-up kits provide high quality sequence data, while they generally have high cost. Here, we describe rapid, effective and low-cost dideoxy terminator clean-up method using a home-made magnetic beads suspension, MagNA, and magnetic separator. We found that MagNA enables rapid and efficient clean-up at ~1/100 of the cost of commercially available kits. The magnetic separator made using low-cost neodymium magnets worked well for the MagNA separation, representing a rapid, efficient and cost-effective dideoxy terminator clean-up system.

## Introduction

Dideoxy/Sanger sequencing has been widely used for DNA sequencing for nearly half a century. The first dideoxy sequencing paper uses an isotopically labeled dideoxy terminator and denaturing polyacrylamide gel electrophoresis [1]; however, a fluorescent dye-labelled dideoxy terminator (e.g., BigDye terminator, Thermofisher) and automated DNA sequencer (e.g., Applied Biosystems 3500XL Genetic Analyzer, Thermofisher) have replaced them, respectively. After the sequencing reaction, the excess dideoxy terminators need to be removed before using the DNA sequencer. For the ‘clean-up’ step, there are several options, each of which has different advantages and disadvantages. Ethanol precipitation is a widely used clean-up method, which requires low costs but a long centrifugation time (~ 1 hr). Furthermore, ethanol precipitation frequently delivers low-quality results. Recently, the BigDye XTerminator purification kit (Thermofisher) has been developed, which outperforms other clean-up methods for sequencing quality, but at a high costs. In addition, this kit needs 30 min of vigorous mixing for complete absorption of the excess dideoxy terminators. Commercially available gel-filtration-based purification kits can enable rapid purification, but also require a high cost. Solid phase reversible immobilization (SPRI) technology [2] is another option for rapid and efficient clean-up of the sequence reaction. However, commercially available magnetic beads that are necessary for the removal of excess dideoxy terminators using SPRI technology are expensive (e.g., CleanSEQ Dye-terminator Removal Kit, Beckman Coulter) and need a specialized magnetic separator for separation of the beads.

Rohland and Reich (2012) reported a home-made SPRI magnetic beads mix (‘MagNA’) for library preparation of NGS by exploiting the capability of MagNA that enables size selection and buffer exchange [3]. MagNA requires ~1/25 of the cost of a commonly used commercial kit (AMPure XP kit, Beckman Coulter) and offers a cost-effective library preparation method. In addition to the use of MagNA in place of AMPure (Beckman Coulter) for NGS library preparation [3], Yoshino et al (2020) recently reported the use of MagNA for RNA extraction from plants [4]. These prompted us to examine if MagNA can be used for other nucleic acid purification purposes including clean-up of the dideoxy sequencing reaction. Here, we report that MagNA can be used for clean-up of dideoxy sequencing reaction in place of the commercial SPRI kit. We also show that a magnetic separator can easily be made by using cheap neodymium magnets (~$1 for 9 magnets) and a pipet tip rack insert. The use of the home-made MagNA SPRI suspension and the magnetic separator represents a rapid, efficient and very low-cost approach for clean-up of the dideoxy sequencing reaction.

## Results and discussion

**Fig 1:**
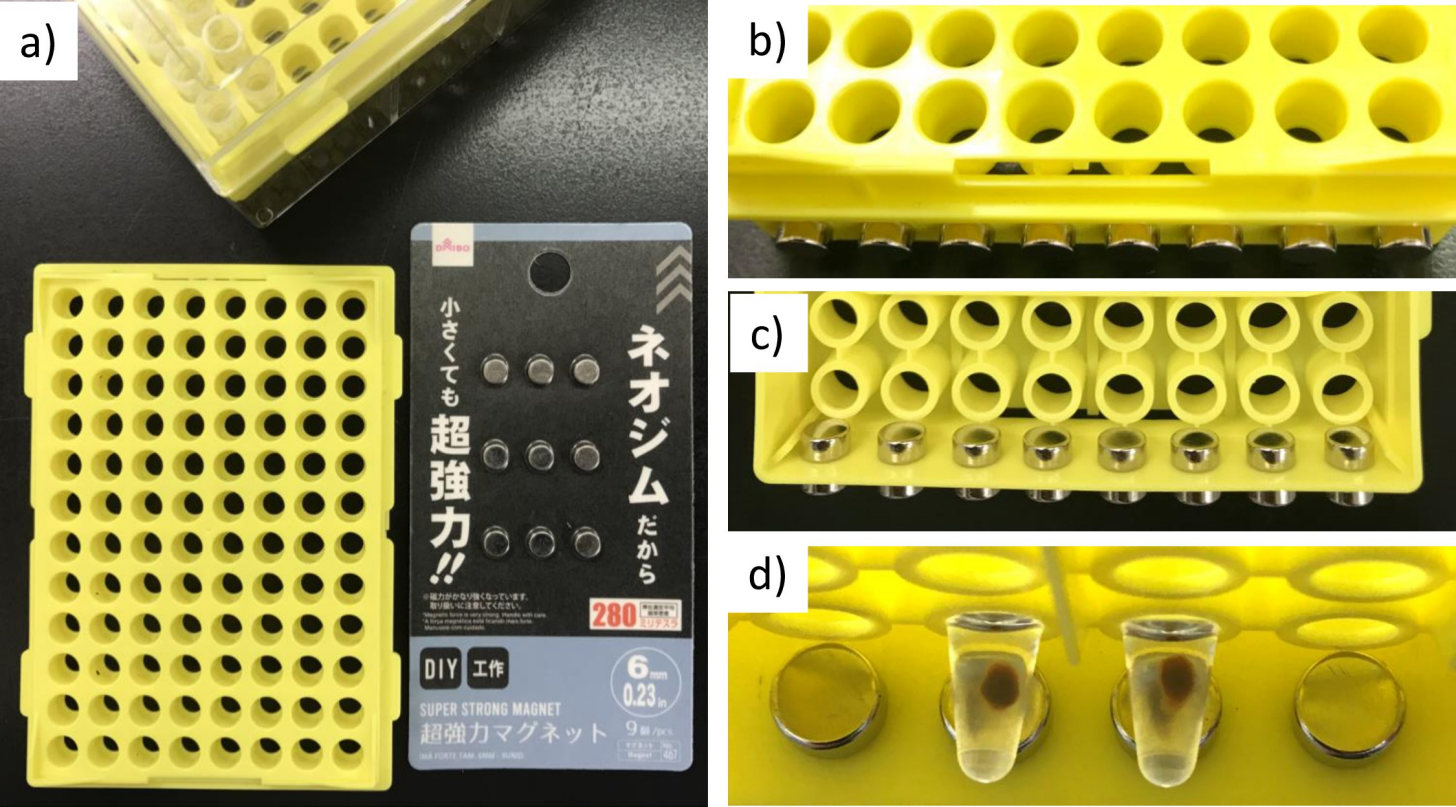
Home-made magnetic separator. (a) Materials for the magnetic separator. (b) Top view of the separator. (c) Bottom view of the separator. (d) Magnetic separation of MagNA.

MagNA was prepared according to Rohland and Reich (2012), which uses 1 ml of SeraMag beads suspension for the preparation of 50 ml of MagNA suspension [3]. Therefore, we expected MagNA will be a cost-effective approach if it can be used for dye-terminator removal. In combination with MagNA, we also tested if neodymium magnets, which are available at a low cost (~$1 for 9 magnets, Fig 1A–1C), can be used for magnetic separation of MagNA. We found that our home-made magnetic separator works well for the magnetic separation of MagNA (Fig 1D). By using the separator, we tested whether the dye terminator removal protocol of the commercially available kit (CleanSEQ) is applicable for MagNA.

**Fig 2:**
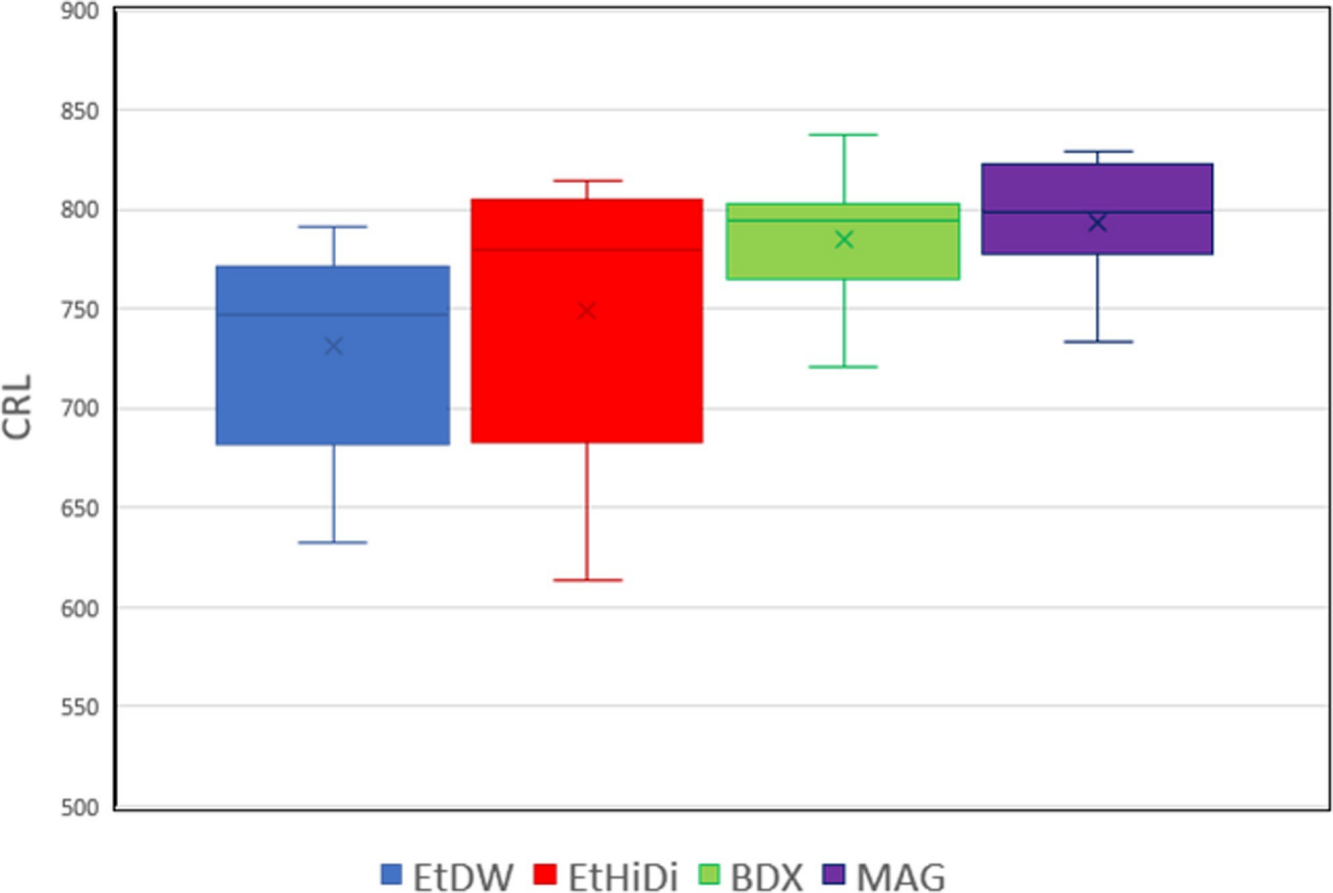
Comparison of sequence data obtained by different clean-up methods. A box plot of CRLs. EtDW: ethanol precipitates suspended by DW, EtHiDi: ethanol precipitates suspended by HiDi formamide, BDX: BigDye XTerminator purification kit, Mag: MagNA purification.

The Contiguous Read Lengths (CRL) of MagNA-purified (Mag) samples were comparable to those of the samples treated using the BigDye XTerminator purification kit (BDX) and significantly longer than ethanol precipitated samples resuspended in DW (EtDW) (Fig 2 and Table 1). Although no significant difference was found between the MagNA-purified samples and ethanol-purified samples resuspend in HiDi (EtHiDi), the F-test showed that the variance of EtHiDi was significantly larger than that of Mag samples (Table 1). These results showed that the sequence quality of MagNA purification is comparable to that of BigDye XTerminator and higher than those of ethanol precipitation methods.

**Table 1:** CRLs (Contiguous Read Length) of samples purified by different methods.

	EtDW	EtHiDi	BDX	Mag
CRL	748	794	801	800
	718	625	782	733
	768	810	799	798
	633	702	810	805
	763	785	774	821
	680	814	837	829
	747	769	737	791
	682	775	792	738
	791	614	721	793
	779	803	797	828
mean	730.9*	749.1	785	793.6
variance	2361.7	5106.9*	1050.4	1014.8

* indicate significant difference from Mag at 5% level by t-test (mean) or F-test (variance).

Ethanol precipitation and BigDye XTerminator purification kit require relatively long processing times, more than 45 min and 30 min, respectively. On the other hand, MagNA purification can be processed in ~10 min, representing a rapid and efficient clean-up method. Additionally, the MagNA process enables dye-terminator removal at a low cost. The commercially available SPRI-based kit (CleanSEQ) costs ~$ 0.7 for a single sample (10 μl beads suspension) ($3,343 for 50 ml suspension, Beckman Coulter), whereas the 10 μl MagNA suspension is 0.8 cent ($599.64 for 15 ml SeraMag, Cytiva). The BigDye XTerminator purification kit cost $0.3~1.4 depending on the product size ($22,890 for 40,000 samples (cat. No. 4376485) and ~$283 for 100 samples (cat. No. 4376486), Thermofisher). MagNA enables rapid and efficient clean-up at ~1/100 of the cost of commercially available kits. Mijatovic-Rustempasic et al (2012) reported a similar magnetic beads-based purification method [5]. They used BioMag beads (Bangs Laboratories, Inc., Fishers, IN, USA), and reported that the purification cost is ~$0.02, which is more than 20 times higher than our MagNA-based method.

The home-made magnetic separator is also a part of the rapid, efficient and cost-effective dideoxy terminator clean-up system. Magnets are set by their magnetic force, and the separator can be quickly made without the need for special tools. This home-made separator is suitable for low- to middle-throughput processing, and for high-throughput separation, a commercial 96-well format separator may be the choice (e.g., SPRIPlate 96R Ring Super Magnet Plate ($980), Beckman Coulter).

MagNA was originally used for library preparation for NGS [3]. Yoshino et al. (2020) reported that MagNA is also useful for RNA extraction from plants [4]. Our study showed dideoxy terminators can effectively be removed by MagNA. The use of MagNA with the home-made magnetic separator offers rapid, efficient and cost-effective methods for different nucleic acid processing processes.

## Materials and methods

### Preparation of MagNA SPRI beads mix

MagNA was prepared according to Rohland and Reich (2012) by combining Carboxyl-modified Sera-Mag Magnetic Speed-beads (Hydrophobic) (Cytiva, cat. #65152105050250) with polyethylene glycol (PEG)/NaCl buffer. Briefly, 1 ml of Sera-Mag beads suspension was washed with TE buffer twice, and filled up to 50 ml to make a suspension in 18% PEG-8000, 1 M NaCl, 10 mM Tris-HCl pH 8, 1 mM EDTA and 0.05% Tween-20. We also added 0.1% ProClin 300 (Sigma-Aldrich) as a preservative. MagNA is stored at 4°C and works for at least for one year.

### Home-made magnetic separator

The insert of a 200 μl tip rack (123R-755CS, Watson, Japan) and neodymium magnets (Magnet no. 467, ⌀ = 6 mm, 280 mT, Daiso, Japan) were used (Fig 1A). Pairs of magnets were set at the edge of the insert by their magnetic force as they line to tip halls (Fig 1B–1D).

### Clean-up of dideoxy sequencing reaction and sequencing

The sequencing master mix containing BigDye terminator ver. 3.1 (Thermofisher), 5x dilution buffer, primer (M13-20) and template (pGEM3Zf(+)) was prepared, divided into four 10 μl solutions, and reacted in a thermal cycler. The reactions were separately subjected to four different clean-up methods, i.e., ethanol precipitation (EtDW or EtHiDi), BigDye XTerminator purification kit (BDX), and MagNA with the home-made magnetic separator (Mag). Ethanol precipitation and BigDye XTerminator purification were conducted according to the manufacture’s protocols (Thermofisher). Ethanol precipitates were suspended by DW (EtDW) or HiDi formamide (EtHiDi). MagNA purification was conducted according to the protocol of the CleanSEQ Dye-terminator Removal Kit (Beckman Coulter), i.e., 10 μl of MagNA and 42 μl (2.077 x vol. of sequencing reaction + MagNA) of 85% ethanol were added to the 10 μl of sequencing reaction and thoroughly mixed. The tube was placed in the home-made magnetic separator and separated for 2~3 min. After removal of the supernatant, 100 μl of 85% ethanol was added to the tube, and the ethanol was removed after >30 sec. The 85% ethanol washing was repeated once. The tube was removed from the separator, and 40 μl of DW was added. After 2~5 min, the tube was placed in the separator, and 35 μl of supernatant was recovered. The recovered solution was subjected to sequencing along with other purified samples (SeqStudio, Thermofisher). The Contiguous Read Length (CRL) outputted from the sequencer was used to evaluate the quality of the sequence data. The sequencing experiments were repeated for ten times. The protocol described in this peer-reviewed article is published on protocols.io, doi.org/10.17504/protocols.io.kqdg3pdzel25/v1 and is included for printing as S1 File with this article.

## Supporting information

S1 File (PDF)
