# Supplementary material for: Rapid, effective and low-cost purification of dideoxy-sequencing reactions by home-made magnetic beads suspension and magnetic separator
Source: PLoS One. 2022 Dec 22;17(12):e0279432. doi: 10.1371/journal.pone.0279432 (PMC9778496; doi:10.1371/journal.pone.0279432)

Rapid, effective and low-cost purification of dideoxy-sequencing reactions by home-made magnetic beads suspension and magnetic separator

Hidenori Sassa*§ and Kota Ikebe§
Graduate School of Horticulture, Chiba University
648 Matsudo, Chiba 271-8510, Japan

*Corresponding author (e-mail: sassa@faculty.chiba-u.jp)

§ These authors contributed equally to this work

Tel: +81-47-308-8967; Fax: +81-47-308-8720

Abstract
Removal of excess dideoxy terminators from the sequencing mix after the enzymatic reaction is a key process affecting the dideoxy/Sanger sequencing quality. Ethanol precipitation may be the most popular clean-up method because of its low costs; however, it takes a long centrifugation time and frequently results in low quality sequence data. Commercially available clean-up kits provide high quality sequence data, while they generally have high cost. Here, we describe rapid, effective and low-cost dideoxy terminator clean-up method using a home-made magnetic beads suspension, MagNA, and magnetic separator. We found that MagNA enables rapid and efficient clean-up at ~1/100 of the cost of commercially available kits. The magnetic separator made using low-cost neodymium magnets worked well for the MagNA separation, representing a the rapid, efficient and cost-effective dideoxy terminator clean-up system.

Preparation of MagNA suspension is based on following publication.
Rohland N, Reich D. Cost-effective, high-throughput DNA sequencing libraries for multiplexed target capture. Genome Res. 2012; 22: 939–946. Doi:10.1101/gr.128124.111

<u>Chemicals for MagNA suspension</u>
·Ethylenediaminetetraacetic acid (EDTA)

·Sodium chloride (NaCl)
·Polyethylene glycol 8,000 (PEG 8,000)
·Tris(hydroxymethyl)aminomethane hydrochloride (TRIS-HCl)
·ProClin 300 (Sigma-Aldrich)
·Distilled Water (DW, Milli-Q)

Other solutions
85 % Ethanol
10x TE buffer (100 mM TRIS-HCl, pH 8, 10 mM EDTA)

MagNA suspension
·Prepare 50 ml buffer without magnet beads as follow. Final concentration of each chemical is indicated in parenthesis.
Dissolve 9 g PEG 8,000 (18 %), 2.92 g NaCl (1 M), 5 ml 10x TE (1x), 25 μl Tween-20 (0.05 %) and 50 μl ProClin 300 (0.1 %) in DW to prepare 50 ml PEG-NaCl buffer.

·Mix thoroughly Carboxyl-modified Sera-Mag Magnetic Speed-beads (Hydrophobic) (Cytiva, cat. #65152105050250), take 1 ml Sera-Mag suspension and separate the beads by using magnetic separator. Add 1 ml 1x TE, mix, stand on magnetic separator, and remove the supernatant. Repeat this wash once.
·Add 1 ml PEG-NaCl buffer, mix and transfer the suspension to the PEG-NaCl buffer tube to prepare MagNA suspension.
·Store at 4 °C at least for one year.

Materials for home-made magnetic separator
·Neodymium magnets (Magnet no. 467, ⌀ = 6 mm, 280 mT, Daiso, Japan)
·Insert of 200 μl tip rack (123R-755CS, Watson, Japan) (Fig. 1a)

Home-made magnetic separator
·Set pairs of magnets at the edge of the insert by their magnetic force as they line to tip halls (Fig. 1b and c).

Clean-up of dideoxy sequencing reaction by MagNA and home-made magnetic separator
The procedure is based on the protocol of CleanSeq (Beckman Coulter) as below.

·Shake MagNA to fully resuspend the magnetic beads.

·To the 10 µl sequencing reaction, add 10 µl MagNA suspension.
·Add 42 µl 85 % EtOH (Volume of 85% Ethanol = 2.077 × (10 µL + Sequencing Sample Volume) and mix thoroughly.
·Place the tube in the home-made magnetic separator for 2~3 min (Fig. 1d).
· Remove the supernatant, add 100 µl of 85 % ethanol, and remove the supernatant after >30 sec. Repeat the step once.
·Remove the tube from the separator, and add 40 µl DW.
·Place the tube in the home-made magnetic separator for 2~3 min, recover 35 µl of supernatant and subject it for sequencing.

Figure 1. Home-made magnetic separator
(A) Materials for the magnetic separator. (B) Top view of the separator. (C) Bottom view of the separator. (D) Magnetic separation of MagNA.

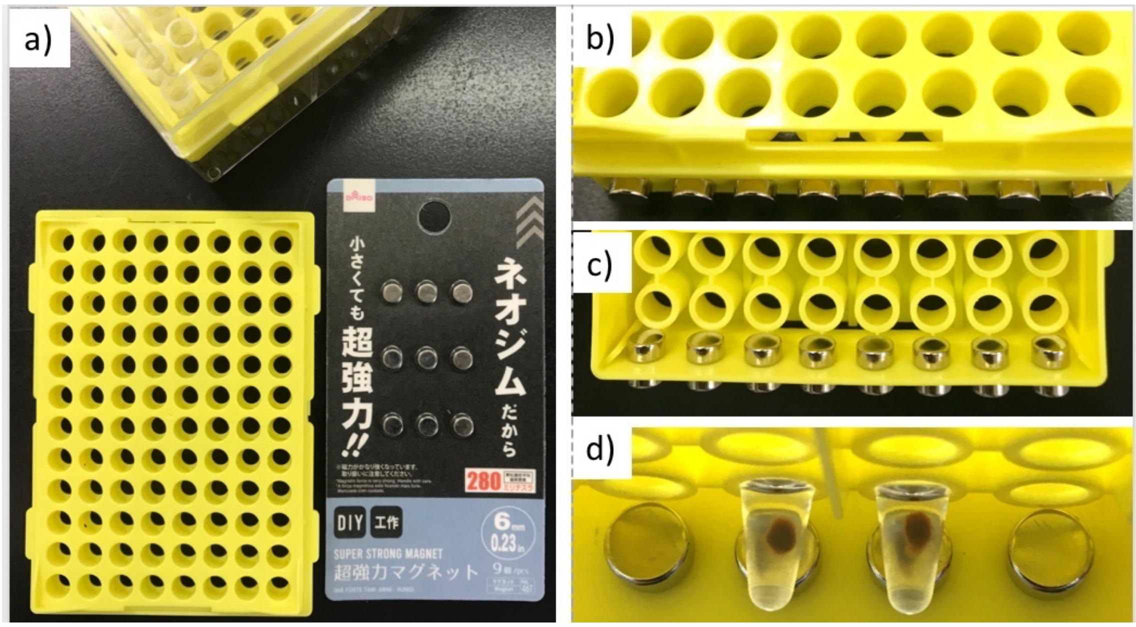

Supplement: S1 File — (PDF) [file pone.0279432.s001.pdf]
